# Prevalence of Bacterial Pathogens Isolated from Canines with Pyoderma and Otitis Externa in Korea: A Systematic Review and Meta-Analysis

**DOI:** 10.3390/vetsci11120656

**Published:** 2024-12-16

**Authors:** Maryum Tanveer, Eurade Ntakiyisumba, Fabrice Hirwa, Hakyoung Yoon, Sang-Ik Oh, Chongchan Kim, Mi Hye Kim, Ji-Seon Yoon, Gayeon Won

**Affiliations:** 1 College of Veterinary Medicine and Bio-Safety Research Institute, Jeonbuk National University, Iksan Campus, Gobong-ro 79, Iksan 54596, Republic of Korea; maryum@jbnu.ac.kr (M.T.); euladus@jbnu.ac.kr (E.N.); fabugih@jbnu.ac.kr (F.H.); hyyoon@jbnu.ac.kr (H.Y.); sioh@jbnu.ac.kr (S.-I.O.); 2 Korea Thumb Vet Co., Ltd., 470-15 Seonhwa-ro, Iksan 54631, Republic of Korea; jongchan1983@hanmail.net; 3 College of Korean Medicine, Woosuk University, Wanju 55338, Republic of Korea; kimmh526@woosuk.ac.kr

**Keywords:** dog, pyoderma, otitis externa, bacterial pathogens, systematic review, meta-analysis

## Abstract

Bacterial skin infections, particularly pyoderma and otitis externa, are widespread in dogs, primarily caused by *Staphylococcus* and *Pseudomonas* species. This study evaluates the prevalence and types of bacterial pathogens in affected dogs in South Korea using a meta-analytical approach. Following the PRISMA guidelines, five electronic databases were searched for relevant studies published between 1990 and 2024. Three researchers independently performed data extraction and quality assessment. A subgroup analysis explored the variability in pathogen prevalence across studies based on bacterial genus, bacterial species, publication year, sampling year, sampling location, infection type, diagnostic method, and sample size. Publication bias was evaluated using funnel plots and Egger’s regression test, with all analyses conducted using the R program. Of the 944 articles, 29 met the eligibility criteria. The pooled bacterial prevalence among infected dogs was 99.95% (95%CI: 99.85–100). *Staphylococcus* was the most prevalent genus (95.93%), followed by *Pseudomonas* (48.43%), *Enterococcus* (20.32%), and *Escherichia* (17.63%). The most common species were *Staphylococcus pseudintermedius* (78.89%), *Staphylococcus intermedius* (71.43%), and *Pseudomonas aeruginosa* (46.13%). This study underscores the need for comprehensive treatment strategies targeting Gram-negative and Gram-positive bacteria, emphasizing further research on antimicrobial resistance patterns and treatment efficacy to enhance canine health outcomes in South Korea.

## Simple Summary

Pyoderma and otitis externa are common skin and ear infections in dogs that cause discomfort through inflammation and itching. These conditions result from multiple factors, including allergies, parasites, and skin disorders, which often lead to secondary bacterial infections, primarily involving *Staphylococcus* and *Pseudomonas* species. While some bacteria, such as *S. intermedius*, are naturally present on canine skin, others, such as *S. pseudintermedius* and *P. aeruginosa*, are environmental and usually absent in healthy dogs. Treatments often include antibiotics, such as amoxicillin-clavulanic acid, cephalexin, and topical agents, such as mupirocin. However, increasing multidrug resistance strains, notably methicillin-resistant *S. pseudintermedius* (MRSP), complicates treatment and increases the need for precise bacterial identification and tailored therapies. Despite research on Korean canine populations, variability in study design has led to fragmented data on bacterial prevalence under these conditions. This systematic review and meta-analysis aimed to fill this knowledge gap by providing consolidated data on the prevalence and types of bacterial pathogens in Korean dogs with pyoderma and otitis externa. By synthesizing existing studies, this analysis provides a clearer understanding of bacterial profiles, supports better-targeted treatments, and informs future research on antimicrobial resistance in veterinary dermatology.

## 1. Introduction

Pyoderma and otitis externa are prevalent dermatological conditions among canine populations, causing significant discomfort through inflammation and pruritus of the skin and outer ear canal [1]. These conditions are primarily caused by bacterial infections, with *Staphylococcus* and *Pseudomonas* species being the most predominant [2,3]. While some bacterial species, such as *Staphylococcus intermedius* and *Staphylococcus epidermidis,* are part of the natural microflora of canine skin, others, including *Staphylococcus pseudintermedius*, *Pseudomonas aeruginosa*, and *Enterococcus* spp., which are mostly isolated from positive subjects, are typically acquired from the environment and do not normally thrive on healthy skin [4]. Clinical signs of canine pyoderma include primary lesions such as papules and pustules, followed by secondary lesions like crusting, epidermal collarettes, alopecia, scaling, erythema, pruritus, lichenification, and hyperpigmentation [5,6]. While the clinical signs of otitis externa include inflammation, ear scratching, head shaking, and ear discharge, prolonged disease could cause symptoms such as offensive odor, edema, erythema, and pruritus [7,8]. The etiology of these conditions is multifactorial, involving an interplay of predisposing, primary, secondary, and perpetuating factors. Primary factors, including allergies (environmental or food-related), ectoparasites (fleas, mites, and lice), and keratinization disorders, often initiate an inflammatory response [9]. Systemic medications such as amoxicillin-clavulanic acid, cephalexin, enrofloxacin, and marbofloxacin, along with topical therapies such as mupirocin and bacitracin, are often recommended for managing moderate to severe cutaneous and subcutaneous infections in dogs [10,11]. However, the growing incidence of multidrug resistance (MDR) in bacterial pathogens has significantly complicated the treatment of associated infections [12]. For instance, *Staphylococcus pseudintermedius*, a key pathogen in canine dermatitis, has demonstrated a notable rise in methicillin-resistant strains among veterinary patients over the last decade, further challenging empirical treatment approaches [13,14]. Additionally, increasing resistance to fluoroquinolones has been observed in *S. intermedius* isolates from cases of canine pyoderma and otitis externa [15]. Understanding the specific etiological agents involved in these conditions is thus crucial for guiding effective treatment and prevention strategies. 

Although previous studies have offered valuable insights into pyoderma and otitis externa in canine populations across various countries, they vary in scope, sample size, and findings, leading to a fragmented understanding of the bacterial landscape associated with these conditions. Recent reports indicate a rise in canine skin and soft tissue infections, including otitis externa and pyoderma, caused by bacterial pathogens, particularly methicillin-resistant *Staphylococcus pseudintermedius* (MRSP), in South Korea. This trend poses significant challenges to veterinary and public health, underscoring its importance as a critical area of study [16,17]. Despite the importance of this issue, no systematic attempt has been made to synthesize data on the prevalence of bacterial pathogens in dogs with pyoderma and otitis externa, particularly in Korea. This systematic review and meta-analysis aimed to address this gap by integrating the current data and providing more robust estimates of the prevalence of bacterial pathogens isolated from dogs infected with dermatitis and otitis externa in Korea. By systematically reviewing the existing literature and performing a meta-analysis, this study seeks to provide a clearer picture of the bacterial etiology of these conditions, offering valuable insights for both clinical practice and future research in veterinary dermatology and microbiology.

## 2. Methodological Approach

### 2.1. Research Outline

In this study, the prevalence of bacterial pathogens isolated from dogs infected with pyoderma and otitis externa across Korea was estimated by adhering to the “Preferred Reporting Items for Systematic Reviews and Meta-Analyses Protocol” (PRISMA-P) guidelines [18] (Table S1). The study objective was constructed in the “population, exposure, comparator, and outcome” (PECO) format [19], in which “population” indicated infected canine samples and “exposure” referred to bacterial pathogens. As the focus of this study was on prevalence, the “comparator” element was not applicable and was therefore excluded. The total number of infected dogs and the number of dogs testing positive for bacterial pathogens were recorded as the “outcome” in each primary study.

### 2.2. Database Search

Five electronic databases (PubMed, Scopus, Web of Science, Embase, and ScienceON) were searched to locate related articles published between 1 January 1990 and 31 July 2024, by developing an appropriate search string (mentioned in Table 1). All references were organized using Endnote X9 software, and duplicate studies were removed after the import process. The bibliography of the downloaded articles was also reviewed manually to identify any potentially missed studies from the initial search [20,21].

### 2.3. Inclusion Criteria and Selection Process

Three individual researchers evaluated the retrieved articles based on predefined inclusion and exclusion criteria [22] outlined in Table 1. Any disagreements were resolved through consensus or arbitration by a separate reviewer. The process began with a review of titles and abstracts, followed by the evaluation of full texts. Articles meeting the required criteria were selected for the meta-analysis [23,24]. 

### 2.4. Quality Evaluation Using the GRADE Approach

The quality of selected studies was assessed using criteria based on the Grading of Recommendations Assessment, Development, and Evaluation (GRADE) approach [25,26]. The following criteria were checked for data scoring: (1) a clearly described diagnostic method, (2) a clearly described sampling method, (3) an indication of the sampling year, (4) random sampling, and (5) an indication of essential characteristics of the study subjects (age, sex). According to these criteria, each article was awarded one point for each satisfied criterion, and the points were summed to obtain the article’s total score. Based on this score, each publication was classified as high (score = 4 or 5), medium (score = 3), or low quality (score = 0 to 2). 

### 2.5. Extraction of Data

Following the validation of eligible studies for quality, relevant information was gathered and organized in a Microsoft Excel spreadsheet to support data analysis. The extracted data included several key variables such as author name, publication year, sampling year, sampling location, dog’s status (stray, pet, rescued), dog’s age, dog’s sex, type of preceding infection (pyoderma, otitis externa, or both), sample type (pus, blood, swab), sampling strategy, diagnostic method (biochemical tests, or PCR), isolated bacteria (genus, species), sample size (total number of infected subjects), and number of samples positive for bacteria. If a study included multiple trials (e.g., categorized by infection type or identified bacteria), data were extracted separately for each trial.

### 2.6. Quantitative Analysis and Data Processing

To address the expected heterogeneity in this meta-analysis, a random-effects model was employed to provide a more robust estimate of the overall effect size. This approach accounts for within-study and between-study variations, making it appropriate for combining data from diverse sources [27]. To ensure the stability of variances and arrange the data under a normal distribution, prevalence estimates from individual studies were transformed using a logit function [28] by applying the following formula:

(1) $$logit\;p=\ln\left(\frac{p}{1-p}\right),$$

(2) $$\mathrm{with}\;\mathrm{variance}:\;var\left(logit\;p\right)=\frac{1}{\mathrm{np}}+\frac{1}{n\left(1-p\right)},$$

where “*n*” is the total sample size and “*p*” is the prevalence of the pathogen under study. The transformed data were then analyzed using a Generalized Linear Mixed Model (GLMM), with between-study variance (τ^2^) estimated using the Maximum Likelihood (ML) method [29]. For clearer interpretation, prevalence rates and their corresponding 95% confidence intervals (CIs) were back-transformed from the logit scale to their original percentage values. The degree of between-study heterogeneity was evaluated using the Q test and *I^2^* statistics, which quantify the proportion of variability in effect estimates attributable to true heterogeneity rather than chance. A *p*-value < 0.05 from the Q test and an *I^2^* value exceeding 50% were considered indicative of substantial heterogeneity. To explore the sources of this heterogeneity, several pre-specified covariates—bacterial genus, bacterial species, publication year, sampling year, sampling location, infection type, diagnostic method, and sample size—were analyzed by subgroup and meta-regression analyses for their association with variations in prevalence rates. Additionally, the prevalence of MRSP was also quantified by meta-analysis, given its clinical importance in guiding empirical therapy. To assess the robustness of the meta-analysis findings, a sensitivity analysis using the leave-one-out method was conducted to identify any outliers or influential studies that could potentially impact the pooled prevalence estimate [30,31]. All statistical analyses were performed using R software (*version 4.1.2*) in combination with R Studio (*version 1.4.1106*), employing specific R packages such as “metafor” (*version 3.8-1*), “meta” (*version 5.5-0*), “MuMIn” (*version 1.43.17*), and “dmetar” (*version 0.0.9000*).

### 2.7. Reporting Bias in Published Literature

Bias in published literature indicates the tendency to selectively publish studies based on the statistical significance, size, and direction of their results [32]. This can pose a significant threat to systematic reviews and meta-analyses by misrepresenting effect size estimates. In this study, the potential for publication bias in the prevalence of bacterial pathogens in dogs with pyoderma and otitis externa across Korea was assessed through visual examination of the symmetry of a contour-enhanced funnel plot [33]. To minimize subjective interpretation and improve accuracy, publication bias was quantitatively assessed using Egger’s regression test [34]. Additionally, the trim-and-fill method was applied to adjust for bias by imputing missing studies into the funnel plot, ensuring a more accurate effect size estimate [33].

## 3. Results

### 3.1. Database Search and Article Selection

**Figure 1:**
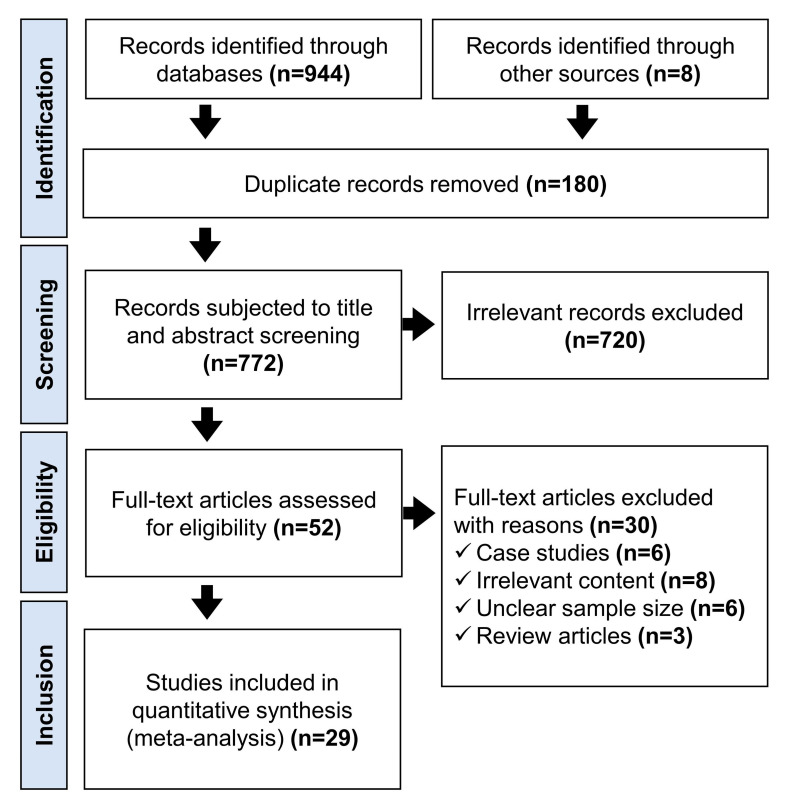
Study selection process for the systematic review and meta-analysis (PRISMA flow chart).

A comprehensive search of the electronic databases was performed in accordance with the PRISMA guidelines to ensure transparent reporting of the research process. The flow of the search and selection process is illustrated in Figure 1, showing the number of articles collected at each stage. The initial search across databases and other sources resulted in 952 documents. After removing duplicates, 772 records remained. Following the screening of titles and abstracts, 720 studies were excluded, and an additional 30 documents were removed due to irrelevance during the full-text screening. Eventually, 29 articles were deemed eligible for inclusion in this meta-analysis.

### 3.2. Quality Assessment

Based on the quality assessment criteria, most of the included studies were classified as either high or medium quality (Table S2). A total of twenty-four studies [15,17,35,36,37,38,39,40,41,42,43,44,45,46,47,48,49,50,51,52,53,54,55,56] were categorized as high quality as they satisfied four or more of the predefined criteria. These studies provided clear descriptions of diagnostic methods, sampling strategies, sampling methods, and sampling year and indicated the essential characteristics of the study subjects, such as age and/or sex, ensuring a comprehensive understanding of the study characteristics. The other five studies [57,58,59,60,61] were rated as medium quality as they fulfilled three of the five criteria. Although these studies did not provide detailed information on certain aspects, such as characteristics of the study subjects, such as age and sex, they offer other valuable insights, such as clarity of sampling and diagnostic characteristics. Overall, all studies fulfilled three to four of the five quality criteria and were therefore warranted to be good quality and suitable for meta-analysis. Importantly, no studies were classified as low quality (having scores of 0–2), so it is believed that none of the included studies negatively impacted the overall findings of the meta-analysis.

### 3.3. Descriptive Characteristics of Eligible Studies

**Figure 2:**
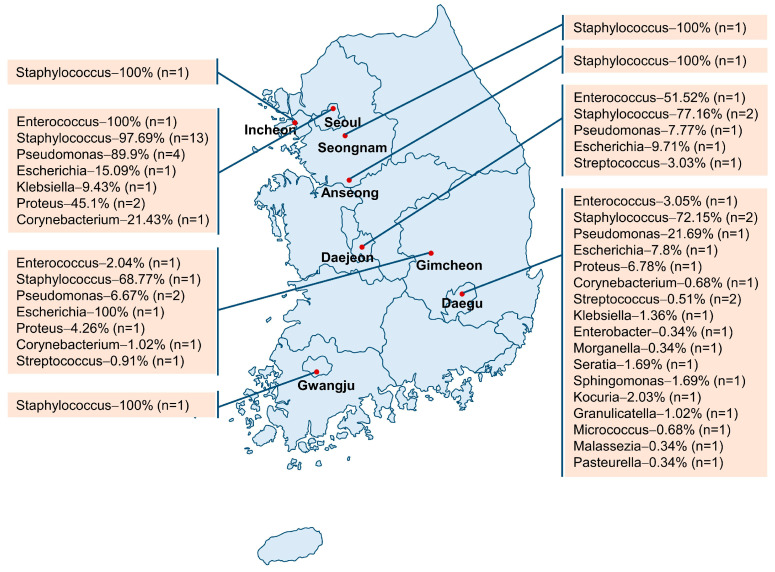
The sampling locations of canine patients with the reported prevalence of diagnosed bacteria (n represents the number of studies).

This meta-analysis included 29 cross-sectional epidemiological studies investigating the prevalence of bacterial pathogens in dogs with pyoderma and/or otitis externa that visited veterinary hospitals across Korea between 2002 and 2021 (Table S3). The samples were collected from various locations across Korea, including Gwangju, Seoul, Daejeon, Daegu, Seongnam, Anseong, Gimcheon, and Incheon. Figure 2 presents a map of these locations along with the prevalence of the diagnosed bacteria. Of the 29 studies, 6 collected samples from dogs infected with pyoderma, 11 from dogs with otitis externa, and 12 studies collected samples from dogs with both conditions. A total of 17 bacterial genera and 49 species were identified in the collected samples (Table 2). In all the studies, samples were obtained by swabbing the skin and/or ears of the dogs. For bacterial identification, 26 of the 29 studies employed PCR assays (genotypic identification), whereas the remaining 3 studies relied only on biochemical tests (phenotypic identification) to diagnose the type of bacteria. The infected dogs were primarily pets, ranging in age from less than 1 year to 15 years, with both sexes being represented (Table S3).

### 3.4. Statistical Analysis

**Figure 3:**
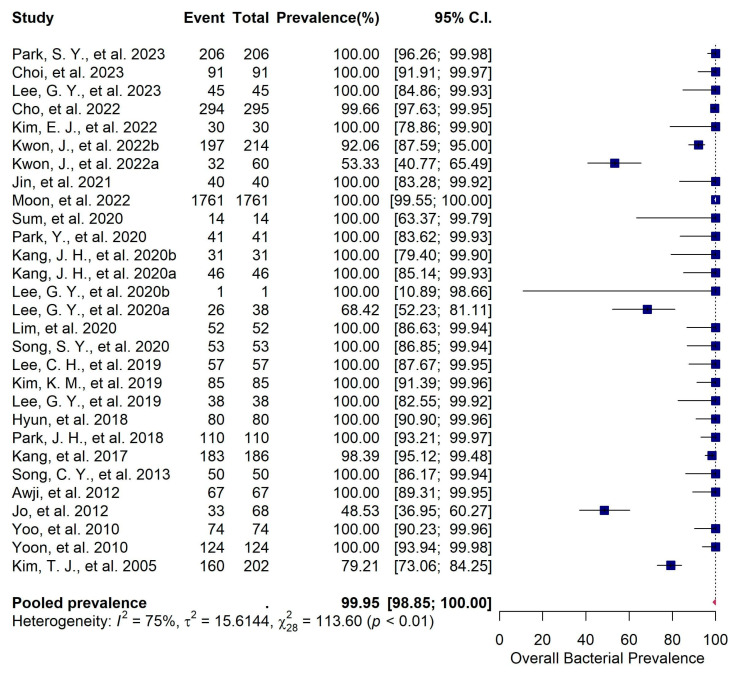
Forest plot illustrating the meta-analysis of 29 studies on the prevalence of bacterial pathogens in dogs infected with pyoderma and otitis externa. (Detailed information on the studies included in this figure is provided in Table S3).

**Figure 4:**
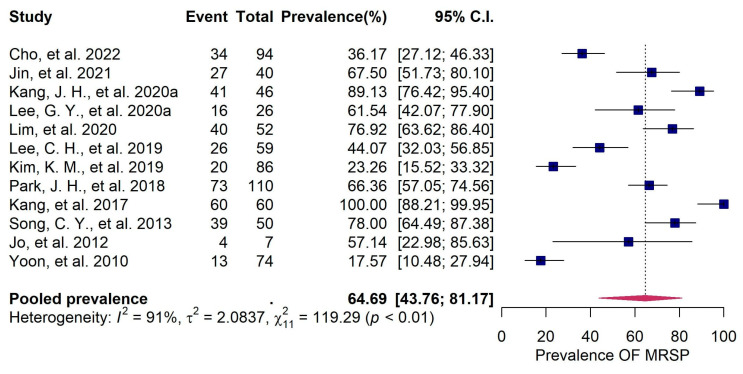
Forest plot illustrating the meta-analysis of 12 studies on the prevalence of methicillin-resistant *Staphylococcus pseudintermedius* (MRSP) in dogs infected with pyoderma and otitis externa. (Detailed information on the studies included in this figure is provided in Table S4).

The 29 primary studies that reported the prevalence of bacterial pathogens in canine patients infected with pyoderma and/or otitis externa were evaluated using a random effects meta-analysis. The estimated pooled bacterial prevalence was 99.95% (95%CI: 98.85–100), suggesting that bacterial pathogens play a major role in canine pyoderma and otitis externa in Korea (Figure 3). Despite the high heterogeneity (*I^2^* = 75%), the sensitivity analysis, conducted using the leave-one-out method, identified no outlier studies and confirmed that the prevalence estimates ranged from 97% (95% CI: 95–99) to 98% (95% CI: 96–99) (Figure S1), indicating that the results of the meta-analysis are robust and reliable. Of the 29 eligible studies, 12 provided data on the antimicrobial resistance of *S. pseudintermedius* to oxacillin. Oxacillin was selected as a reliable and standardized indicator for detecting methicillin resistance because it targets the same penicillin-binding proteins as methicillin, and the genetic determinants of resistance to both antibiotics (the *mecA* or *mecC* genes) are the same in staphylococcal species [62]. Additionally, oxacillin is preferred over methicillin in susceptibility testing due to its stability and reproducibility in laboratory settings [63]. A meta-analysis of these 12 studies indicated a pooled prevalence of 64.69% (95%CI: 43.76–81.17) for MRSP (Figure 4).

**Figure 5:**
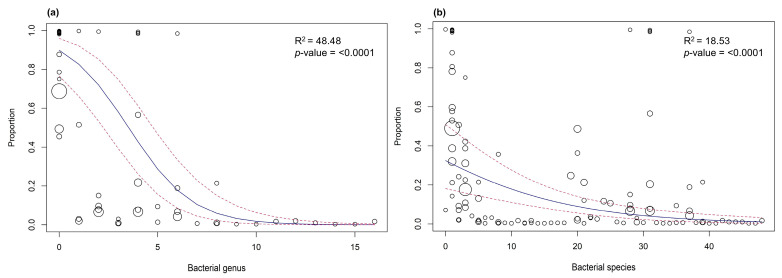
Meta-regression of study trials on the prevalence of bacterial pathogens in dogs infected with pyoderma and otitis externa based on bacterial genus (**a**) and species (**b**). The solid lines (blue) represent the regression illustrating the relationship between the bacterial genus/species and prevalence estimates, while the dashed lines (red) indicate the 95% confidence intervals (CIs) of the regression model. The circles represent individual study trials in the meta-regression, with their size indicating their weight.

Subgroup analysis based on bacterial genus revealed that Staphylococcus was the most prevalent (95.93%, 95%CI: 92.19–97.92) among the canine patients, followed by Pseudomonas (48.43%, 95% CI: 24.94–72.64), Enterococcus (20.32%, 95%CI: 6.46–48.5) and Escherichia (17.63%, 95% CI: 6.39–40.14) (Table 2). Subgroup analysis for bacterial species showed that *S. pseudintermedius* (78.89%, 95%CI: 67.2–87.21) and *S. intermedius* (71.43%, 95%CI: 20.11–96.13) were the predominant species, followed by *S. felis* (24.76%, 19.34–31.11), *P. aeruginosa* (46.13%, 95%CI: 24.17–69.7), *E. coli* (16.99%, 6.49–37.64), *E. faecalis* (11.82%, 95%CI: 4.11–29.54), and *E. faecium* (7.04%, 95%CI: 1.75–24.29) (Table 2). Subgroup analysis based on sampling location showed that samples taken from canine patients in Gimcheon (99.88%, 95%CI: 96.59–100), Daegu (99.52%, 95%CI: 89.54–99.98), and Seoul (98.33%, 95%CI: 95.38–99.41) exhibited the highest bacterial prevalence compared to other locations. Moreover, subgroup analysis categorized by infection type indicated that pyoderma patients have a higher bacterial prevalence (98.92%, 95%CI: 94.90–99.78) than otitis externa patients (93.2%, 95%CI: 81.99–97.63). The analyses based on other variables—publication year, sampling year, diagnostic method, and sample size—are summarized in Table 3. Overall, subgroup analyses specify that three of the considered variables, such as bacterial genus, bacterial species, and infection type were statistically associated with the between-study heterogeneity (*p*-value < 0.05), while others were not (Table 2 and Table 3). Meta-regression analysis also indicated that the covariates bacterial genus and bacterial species were statistically associated with the between-study heterogeneity (*p*-value < 0.0001) and contributed 48.48% and 18.53% in between-study heterogeneity (Figure 5). For other variables, including publication year, sampling year, sampling location, infection type, diagnostic method, and sample size, the meta-regression analysis revealed that their individual contributions to between-study heterogeneity were minimal. However, when considered collectively, these variables accounted for 16.26% of the heterogeneity across prevalence estimates (Table 3).

### 3.5. Publication Bias

**Figure 6:**
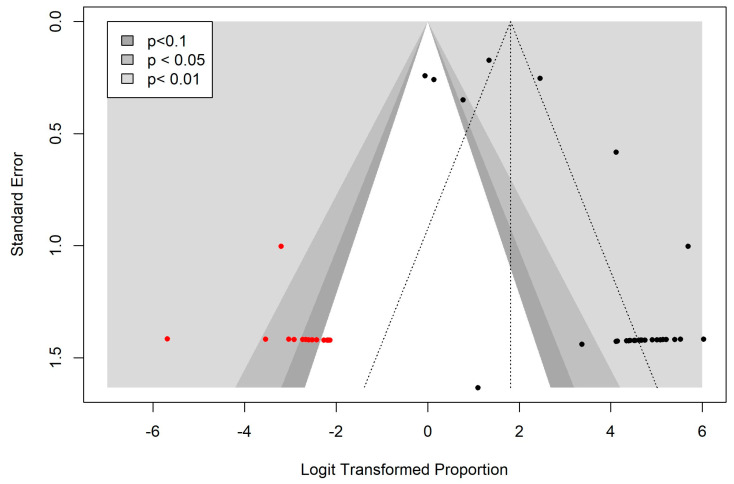
Funnel plot showing publication bias in the prevalence of bacterial pathogens in dogs with pyoderma and otitis externa. Black circles represent the studies included in this meta-analysis, while red circles indicate the studies imputed using the trim-and-fill method.

To assess the degree of bias in the published literature regarding the prevalence of bacterial pathogens in dogs with pyoderma and/or otitis externa in Korea, a contour-enhanced funnel plot was generated. The plot displayed various significance levels (<0.1, <0.05, and <0.01) with log-transformed proportions plotted on the *x*-axis and their respective standard errors on the *y*-axis. The resulting funnel plot exhibited an asymmetric distribution of studies around the mean effect, indicating potential publication bias (Figure 6). This asymmetry was further confirmed by Egger’s regression test, which produced a statistically significant result (*p* < 0.0001), suggesting bias in publication. Using the trim-and-fill method, 14 missing studies were identified, causing a shift in the pooled prevalence estimate from 99.95% (95%CI: 98.85–100) to 85.94% (95%CI: 67.81–94.67). Despite this adjustment, the shift did not originate from the original dataset and, therefore, did not compromise the validity of the findings. These results highlight the need for additional research to improve understanding of the prevalence of bacterial pathogens in infected subjects.

## 4. Discussion

This systematic review and meta-analysis provided a comprehensive assessment of the prevalence and diversity of bacterial pathogens isolated from canine patients affected by pyoderma and otitis externa in Korea. Our findings demonstrate that bacterial pathogens play a critical role in the etiology of these conditions, with an estimated pooled prevalence of 99.95% (95%CI: 95.85–100). This high prevalence underscores the significance of bacterial involvement in dermatological infections among Korean dogs, which is consistent with the conclusions of previous studies that highlighted bacteria as primary agents in canine pyoderma and otitis externa [2,64]. The observed dominance of Staphylococcus species, particularly *S. pseudintermedius* (78.89%, 95%CI: 67.2–87.21) and *S. intermedius* (71.43%, 95%CI: 20.11–96.13) aligns with prior literature that identifies *S. pseudintermedius* as the principal pathogen associated with canine skin infections [65]. Notably, *S. pseudintermedius* is often part of the commensal flora of canine skin but can become opportunistic under conditions of compromised skin integrity, leading to infections [66]. The relatively high prevalence of *S. intermedius* further suggests that *Staphylococcal* species, including non-pathogenic strains, could play a dual role by acting as both harmless residents and opportunistic pathogens under certain conditions [67]. The analysis also revealed that Pseudomonas was the second most prevalent pathogen after Staphylococcus, with *P. aeruginosa* having a pooled prevalence of 46.13% (95%CI: 24.17–69.7). This species is a well-known opportunistic pathogen that is often associated with chronic and severe cases of otitis externa, particularly in dogs with recurrent ear infections [68]. The ability of *P. aeruginosa* to thrive in moist environments and its intrinsic resistance to multiple antimicrobial agents further complicate treatment strategies [69]. Given the substantial presence of this pathogen, especially in cases of otitis externa, veterinarians must be vigilant in the diagnosis and management of chronic infections to prevent the escalation of antimicrobial resistance (AMR).

The emergence of multidrug-resistant (MDR) strains, particularly methicillin-resistant *S. pseudintermedius* (MRSP), poses a growing threat to veterinary practice in Korea. Our study specifically quantifies the prevalence of MRSP (64.69%, 95%CI: 43.76–81.17), indicating that the increasing global incidence of this resistant strain has made the empirical treatment of canine skin infections increasingly challenging [40]. The persistent presence of *Staphylococcus* species combined with their capacity to acquire resistance genes highlights the need for targeted therapeutic approaches and antimicrobial stewardship programs in veterinary care. The identification of *Escherichia coli* (16.99%, 95% CI: 6.49–37.64) and *Proteus mirabilis* (16.32%, 95%CI: 5.39–40.02) as significant contributors to both pyoderma and otitis externa further broadens the bacterial spectrum involved in these infections. Although less frequently implicated than *Staphylococcus* and *Pseudomonas* species, the presence of these enteric bacteria suggests a possible environmental or zoonotic component, particularly in cases where hygiene conditions may be suboptimal. This finding aligns with earlier research suggesting that poor hygiene or environmental contamination may increase the risk of enteric pathogenic in dogs [70].

Another notable aspect of this study is the high heterogeneity observed among the included studies. The significant between-study variance (*I^2^* > 50%) suggests considerable differences in study design, sampling method, and diagnostic approach. These factors likely contributed to the observed heterogeneity in prevalence estimates. For instance, variations in diagnostic techniques ranging from biochemical tests to PCR assays may have influenced the reported prevalence of certain pathogens [71]. Additionally, the geographic diversity of sampling sites across Korea, as well as differences in environmental factors, such as humidity and temperature, could have affected bacterial colonization patterns in canine populations [72]. Although we attempted to account for some of these factors using moderator analyses (Table 2 and Table 3), the complexity of bacterial infections in dogs requires further investigation to delineate the precise environmental and biological drivers of pathogen prevalence.

Assessment of publication bias revealed some degree of asymmetry in the funnel plot, indicating that studies reporting higher prevalence rates were more likely to be published than those with lower prevalence rates (Figure 6). Currently, the tools designed to assess the likelihood of publication bias are mainly for randomized controlled trials (RCTs), and they follow the assumption that the study publication is influenced by its statistical significance [33]. However, studies included in the meta-analysis of prevalence are observational (e.g., cross-sectional studies) and non-comparative studies and thus do not calculate significant levels for their results [34]. Therefore, the available tools to investigate publication bias are generally considered less useful in the context of meta-analyses of prevalence studies and their indication of possible publication bias does not necessarily pose a threat to the validity of the findings and should be interpreted with caution [33,34]. Another meta-analysis study about bacterial prevalence also underscored the importance of assessing publication bias to enhance current knowledge [73]. 

The findings of this study hold significant clinical relevance, highlighting the major roles of Staphylococcus, Pseudomonas, and enteric bacteria in canine skin and ear infections. Although other microorganisms, such as Malassezia yeast, can occasionally contribute to otitis externa, bacteria remain the predominant causative agent [74]. Veterinarians must prioritize accurate microbial diagnosis before initiating treatment. While variability in diagnostic capabilities across clinics may limit the direct implementation of antibiograms, our findings can guide practical solutions. Regional AMR surveillance programs, simplified treatment guidelines based on pooled resistance data, and enhanced access to diagnostic resources through collaborative networks and regional laboratories can help clinicians overcome the current issue of AMR [75]. Additionally, training initiatives for veterinarians can emphasize the importance of using regional antibiogram data to optimize empirical therapy. Furthermore, the high prevalence of MDR pathogens, particularly MRSP, underscores the need for more prudent antibiotic use along with alternative treatment strategies such as the use of topical agents or immunomodulatory therapies. The predominance of Staphylococcus and Pseudomonas species, coupled with the emergence of MDR strains, calls for concerted efforts to improve diagnostic accuracy and antimicrobial stewardship in veterinary practice. For instance, one study reported a 35.7% prevalence of methicillin-resistant Staphylococcus in canine pyoderma, emphasizing the critical role of bacterial pathogens in these infections [76].

Although systematic reviews and meta-analyses provide valuable insights, they have certain limitations that must be acknowledged [77]. For instance, while our meta-analysis provides detailed insights into the bacterial spectrum associated with pyoderma and otitis externa, most included studies lacked detailed information about the dogs’ specific physiological conditions, such as breed, size, or whether they were pet dogs, strays, or used for other purposes. Additionally, ecological variables of the sampling locations, such as temperature, humidity, and urban versus rural settings, were often not reported. Future research should prioritize investigating the environmental, genetic, and physiological factors that predispose certain canine populations to bacterial infections. Additionally, efforts should be directed toward developing innovative therapeutic strategies to address the growing challenge of antimicrobial resistance. Addressing these gaps will enable better management and prevention of these debilitating conditions, ultimately improving dogs’ quality of life and alleviating the burden on veterinary healthcare systems.

## Figures and Tables

**Table 1:** Eligibility criteria and search string for assessing the prevalence of bacterial pathogens isolated from dogs infected with pyoderma and otitis externa in Korea.

Criteria	Inclusion	Exclusion
Study type	Cross-sectional studies	Cohort studies, case–control studies, ecological studies, and secondary research
Population	Dogs with pyoderma or otitis externa	Healthy dogs, other members of the family Canidae, and other animal species
Exposure	Bacterial pathogens	Non-bacterial pathogens (fungi, algae, viruses)
Outcomes	Incidence rate of bacteria	Alternative outcomes (genomic analysis, antibiotic resistance)
Country	South Korea	Countries other than South Korea
**Search string:** (dog OR canine OR companion OR companion dogs OR puppies OR companion animals) AND (otitis externa OR ear infection OR pyoderma OR skin infection OR otitis OR infection OR atopy OR infestation) AND (bacteria) AND (prevalence OR proportion OR occurrence OR presence OR cases OR event OR inciden* OR isolates OR surveillance OR infect*) AND (Korea*).		

**Table 2:** Subgroup analysis of bacterial pathogens in dogs with pyoderma and otitis externa based on bacterial genus and species.

Genus (No. of Studies)	Subgroup Analysis		Species (No. of Studies)	Subgroup Analysis	
	Pooled Prevalence (%) (95% CI)	*I^2^* (*p*-Value)		Pooled Prevalence (%) (95% CI)	*I^2^* (*p*-Value)
*Staphylococcus* (22)	95.93 (92.19–97.92)	90 (<0.01)	*S. intermedius* (2)	71.43 (20.11–96.13)	96 (<0.01)
			*S. pseudintermedius* (20)	78.89 (67.20–87.21)	92 (<0.01)
			*S. aureus* (6)	10.39 (4.19–23.5)	95 (<0.01)
			*S. schleiferi* (8)	23.92 (11.96–42.13)	91 (<0.01)
			*S. capitis* (1)	4.08 (1.02–14.91)	−
			*S. epidermidis* (5)	4.10 (1.41–11.38)	95 (<0.01)
			*S. warneri* (2)	1.05 (0.13–7.96)	61 (0.11)
			*S. cohnii* (1)	3.23 (0.45–19.64)	−
			*S. haemolyticus* (4)	2.11 (0.58–7.38)	94 (<0.01)
			*S. sciuri* (1)	0.55 (0.08–3.77)	−
			*S. lentus* (1)	0.34 (0.05–2.37)	−
			*S. caprae* (1)	1.7 (0.71–4.02)	−
			*S. chromogenes* (2)	0.43 (0.05–3.4)	0 (0.74)
			*S. hominis* (2)	1.09 (0.17–6.6)	8 (0.3)
			*S. lugdunenis* (1)	0.34 (0.05–2.37)	−
			*S. saprophyticus* (1)	0.34 (0.05–2.37)	−
			*S. simulans* (1)	0.55 (0.08–3.77)	−
			*S. hyicus* (1)	0.55 (0.08–3.77)	−
			*S. parasanguinis* (1)	0.55 (0.08–3.77)	−
			*S. felis* (1)	24.76 (19.34–31.11)	−
*Pseudomonas* (7)	48.43 (24.94–72.64)	97 (<0.01)	*P. aeruginosa* (7)	46.13 (24.17–69.7)	97 (<0.01)
			*P.lutea* (1)	0.68 (0.17–2.68)	−
			*P.putida* (1)	0.34 (0.05–2.37)	−
			*P. stutzeri* (1)	0.34 (0.05–2.37)	−
*Enterococcus* (4)	20.32 (6.46–48.5)	98 (<0.01)	*E. faecalis* (4)	11.82 (4.11–29.54)	99 (<0.01)
			*E. faecium* (3)	7.04 (1.75–24.29)	90 (<0.01)
			*E. avium* (2)	3.34 (0.55–17.90)	0 (0.88)
			*E. gallinarum* (1)	2.54 (1.06–5.95)	−
			*E. casseliflavus* (1)	11.68 (7.88–16.96)	−
			*E. hirae* (1)	10.66 (7.05–15.80)	−
			*E. canintestini* (1)	1.02 (0.25–3.97)	−
			*E. durans* (1)	0.51 (0.07–3.51)	−
*Escherichia* (5)	17.63 (6.39–40.14)	89 (<0.01)	*Escherichia coli* (5)	16.99 (6.49–37.64)	89 (<0.01)
*Proteus* (4)	17.01 (5.3–42.87)	93 (<0.01)	*P. mirabillis* (4)	16.32 (5.39–40.02)	93 (<0.01)
*Klebsiella* (2)	3.69 (0.63–18.75)	88 (<0.01)	*K. pneumoniae* (2)	2.92 (0.51–14.97)	90 (<0.01)
			*K. oxytoca* (1)	0.68 (0.17–2.68)	−
*Corynebacterium* (3)	2.47(0.56–10.17)	91 (<0.01)	*C. auriscanis* (3)	2.46 (0.6–9.46)	91 (<0.01)
*Kocurica* (1)	2.04 (0.92–4.47)	−	*K. kristinae* (1)	1.02 (0.33–3.11)	−
			*K. rosea* (1)	1.02 (0.33–3.11)	−
*Sphingomonas* (1)	1.7 (0.71–4.02)	-	*S. paucimobilis*	1.7 (0.71–4.02)	−
*Seratia* (1)	1.7 (0.71–4.02)	−	*S. marcescens* (1)	1.7 (0.71–4.02)	−
*Streptococcus* (3)	1.13 (0.24–5.14)	0 (0.45)	*S. canis* (2)	1.42 (0.24–8.05)	27 (0.24)
			*S. parasanguinis* (1)	0.68 (0.17–2.68)	−
*Granulicatella* (1)	1.02 (0.33–3.11)	-	*G. elegans* (1)	1.02 (0.33–3.11)	−
*Micrococcus* (1)	0.68 (0.17–2.68)	−	*M. luteus* (1)	0.68 (0.17–2.68)	−
*Enterobacter* (1)	0.34 (0.05–2.37)	−	*E. aerogenes* (1)	0.34 (0.05–2.37)	−
*Alloiociccus* (1)	0.34 (0.05–2.37)	-	*A. otitis* (1)	0.34 (0.05–2.37)	−
*Pasteurella* (1)	0.34 (0.05–3.27)	-	*P. canis* (1)	0.34 (0.05–2.37)	−
*Morganella* (1)	0.34 (0.05–2.37)	−	*M. morganii* (1)	0.34 (0.05–2.37)	−
**Pooled prevalence**	29.09 (22.08–37.25)	99 (<0.01)	**Pooled prevalence**	9.64 (7.73–11.96)	97 (<0.01)

**Table 3:** Subgroup analysis and meta-regression of bacterial pathogens in canine patients with pyoderma and otitis externa based on publication year, sampling year, sampling location, infection type, diagnostic method, and sample size.

Covariates	Subgroup	Meta-Analysis					Meta-Regression			
		Random Effects Model					Univariate		Multivariate	
		No. ofStudies	Prevalence (%)	95% CI	*I^2^* (*p*-Value)	* *p_subgroup_*	R^2^ (%)	*p*-Value	R^2^ (%)	*p*-Value
Publication year	Before 2015	6	95.44	80.4–99.07	91 (<0.01)	0.3	6.54	0.2951	16.26	0.7529
	After 2015	23	98.25	95.9–99.26	87 (<0.01)					
Sampling year	Before 2015	8	96.65	87.23–99.19	92 (<0.01)	0.46	3.1	0.4485		
	After 2015	21	98.23	95.6–99.3	87 (<0.01)					
Sampling location	Gwangju	1	79.21	73.06–84.25	−	0.18	1.31	0.5597		
	Seoul	19	98.33	95.38–99.41	85 (<0.01)					
	Daejeon	2	91.47	35.02–99.53	94 (<0.01)					
	Daegu	2	99.52	89.54–99.98	0 (0.46)					
	Incheon	1	100	91.39–99.96	−					
	Anseong	1	68.42	52.23–81.11	−					
	Seognam	1	100	10.89–98.66	−					
	Gimcheon	2	99.88	96.59–100	54 (0.14)					
Infection type	Pyoderma	6	98.92	94.9–99.78	90 (<0.01)	0.03 *	2.33	0.6114		
	Otitis externa	11	93.2	81.99–97.63	91 (<0.01)					
	Both	12	99.02	96.92–99.7	0 (1)					
Diagnostic method	PCR	26	97.89	95.38–99.05	89 (<0.01)	0.8	0.12	0.7977		
	Biochemical tests	3	97.11	77.16–99.7	44 (<0.01)					
Sample size	More than 150	6	98.52	93.33–99.69	93 (<0.01)	0.57	0.73	0.5781		
	Less than 150	23	97.55	94.24–98.98	86 (<0.01)					

* *p*_subgroup_: *p*-value for subgroup differences.

## Data Availability

The original contributions presented in the study are included in the article/Supplementary Material. Further inquiries can be directed at the corresponding author.

## Supplementary Materials

The following supporting information can be downloaded at: https://www.mdpi.com/article/10.3390/vetsci11120656/s1, Table S1: Preferred Reporting Items for Systematic Reviews and Meta-Analyses Protocol (PRISMA-P) 2020 checklist; Table S2: Quality assessment of the included studies in the meta-analysis of the prevalence of bacterial pathogens in dogs with pyoderma and/or otitis externa in Korea; Table S3: Descriptive characteristics of eligible studies included in the meta-analysis of the prevalence of bacterial pathogens in dogs with pyoderma and/or otitis externa in Korea. Table S4: Descriptive characteristics of eligible studies included in the meta-analysis of the prevalence of methicillin-resistant *Staphylococcus pseudintermedius* (MRSP) in dogs affected with pyoderma and/or otitis externa in Korea. Figure S1: Sensitivity analysis of the bacterial prevalence in dogs with pyoderma or otitis externa in Korea. The prevalence estimates (right) and their corresponding 95% confidence intervals were computed by leaving out each study (left).
