# Supplementary figures and images for: Prevalence of Bacterial Pathogens Isolated from Canines with Pyoderma and Otitis Externa in Korea: A Systematic Review and Meta-Analysis
Source: Vet Sci. 2024 Dec 16;11(12):656. doi: 10.3390/vetsci11120656 (PMC11680266; doi:10.3390/vetsci11120656)

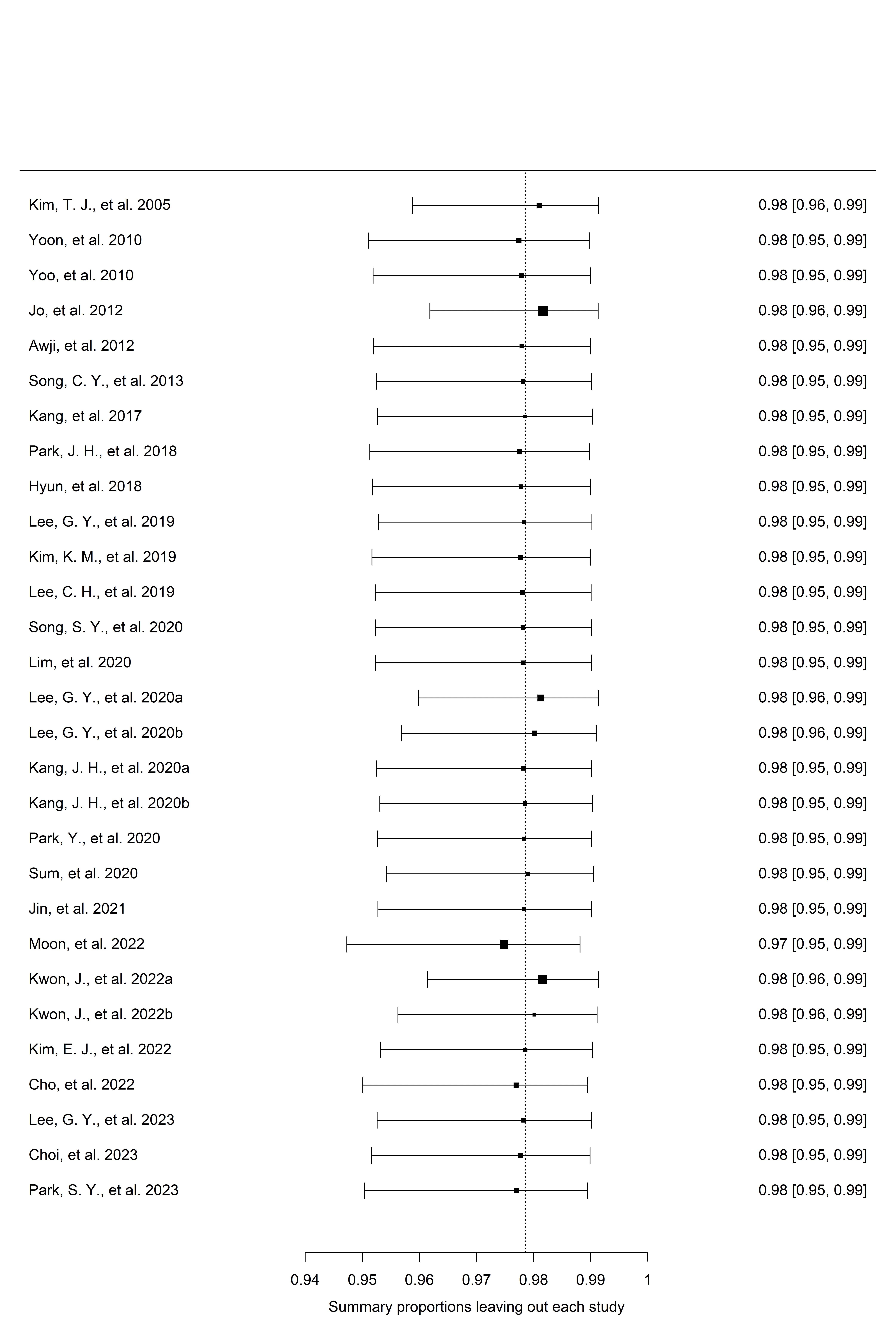

Supplement: Supplementary file 1 [file vetsci-11-00656-s001.zip › vetsci-3323212 - Supplementary material/Figure S1.jpg]
